# A high-precision jujube disease spot detection based on SSD during the sorting process

**DOI:** 10.1371/journal.pone.0296314

**Published:** 2024-01-05

**Authors:** Zhi-Ben Yin, Fu-Yong Liu, Hui Geng, Ya-Jun Xi, De-Bin Zeng, Chun-Jing Si, Ming-Deng Shi

**Affiliations:** 1 College of Information Engineering, Tarim University, Alaer, 843300, China; 2 College of Information Science and Engineering, Xinjiang University of Science & Technology, Korla, 841000, China; 3 Tarim University Library, Tarim University, Alaer, 843300, China; 4 Key Laboratory of Tarim Oasis Agriculture (Tarim University), Ministry of Education, Alaer, 843300, China; Vellore Institute of Technology: VIT University, INDIA

## Abstract

The development of automated grading equipment requires achieving high throughput and precise detection of disease spots on jujubes. However, the current algorithms are inadequate in accomplishing these objectives due to their high density, varying sizes and shapes, and limited location information regarding disease spots on jujubes. This paper proposes a method called JujubeSSD, to boost the precision of identifying disease spots in jujubes based on a single shot multi-box detector (SSD) network. In this study, a diverse dataset comprising disease spots of varied sizes and shapes, varying densities, and multiple location details on jujubes was created through artificial collection and data augmentation. The parameter information obtained from transfer learning into the backbone feature extraction network of the SSD model, which reduced the time of spot detection to 0.14 s. To enhance the learning of target detail features and improve the recognition of weak information, the traditional convolution layer was replaced with deformable convolutional networks (DCNs). Furthermore, to address the challenge of varying sizes and shapes of disease spot regions on jujubes, the path aggregation feature pyramid network (PAFPN) and balanced feature pyramid (BFP) were integrated into the SSD network. Experimental results demonstrate that the mean average precision at the IoU (intersection over union) threshold of 0.5 (mAP@0.5) of JujubeSSD reached 97.1%, representing an improvement of approximately 6.35% compared to the original algorithm. When compared to existing algorithms, such as YOLOv5 and Faster R-CNN, the improvements in mAP@0.5 were 16.84% and 8.61%, respectively. Therefore, the proposed method for detecting jujube disease spot achieves superior performance in jujube surface disease detection and meets the requirements for practical application in agricultural production.

## Introduction

The Xinjiang Province has become an important center for the production and processing of jujube. By the end of 2022, there was a vast cultivation area of 4.13 X 105 square meters dedicated to jujube farming, resulting in an annual production of 3.81 X 106 tons [1]. This development has not only optimized the industrial structure of rural areas but also significantly contributed to the income of local farmers [2]. However, the region has been experiencing significant climatic changes in recent years, with increasing temperatures and precipitation becoming more abundant. These changes pose challenges for jujube farming, particularly for delicate and sweet jujubes. Cold and humid nights make jujubes susceptible to issues such as cracking and black spot disease, which harm their quality and yield [3]. Black spot disease has been identified as the primary issue affecting jujube quality and yield in the northwestern region over the past decade. On average, this results in an annual yield reduction of over 30%, and in some cases, it even exceeds 50%. Jujube black spot disease, primarily caused by fungi such as Alternaria alternata, diminishes fruit quality and, when uncontrolled, may lead to the presence of chemical residues from pesticide use, potentially affecting human health [4]. To ensure marketability and processing quality, it is crucial to identify and remove jujubes containing black spots during the screening process. This step plays a vital role in maintaining the overall quality and market value of jujubes, given the health risks associated with the harmful compounds produced by the bacterium.

The current practice of sorting jujubes for black spot disease involves manual inspection of surface defects before they are made available on the market or processed. However, this approach is time-consuming and subjective, leading to a slow and inaccurate sorting process. Inaccurate sorting directly affects the subsequent processing and sale of jujubes, while slow sorting negatively impacts jujube sorting productivity [5–7]. Timely and accurate identification of plant diseases is essential for enhancing the growth of the fruit and vegetable industries, making it a significant area of research in agricultural development. Various spectroscopic and imaging techniques have been explored for plant disease identification [8–12]. NK Mahanti et al. [11] presented a comprehensive discussion on detection systems including the bio-speckle technique, X-ray imaging, hyperspectral imaging, and thermal imaging. However, these approaches require preliminary data processing and rely on sophisticated and costly instrumentation, making them impractical for widespread deployment. Additionally, this method may not be efficient for disease identification. With the rapid progress in hardware devices, the widespread adoption of machine learning for automatic plant disease detection has become prevalent. Elangovan et al. [13] used a series of steps, including loading, preprocessing, segmentation, feature extraction, and support vector machine (SVM) classification of plant disease images to identify different disease categories. However, machine learning approaches often require complex data preprocessing, and dividing the detection process into multiple tasks can make the overall process more intricate.

In recent years, convolutional neural networks (CNNs) [14] have gained popularity for plant disease identification, owing to advancements in deep learning. Researchers have explored the use of CNNs for plant disease identification [15]. However, early CNN models were limited to identifying the presence of diseases in an image without being able to pinpoint the exact location of the disease [5]. To address this limitation, this study proposes combining target detection algorithms with disease diagnosis applications to identify the type of disease and locate the affected areas. By using deep learning-based plant disease detection methods [16], the diagnosis time in large production areas is reduced, and losses caused by manual diagnosis errors are minimized [17]. There are two main types of target detection methods: two-stage and one-stage methods [18]. Two-stage methods train CNNs for classification based on candidate regions generated by the algorithm, such as Faster R-CNN [19] and R-FCN [20]. However, most of these methods rely on single-scale feature maps for disease prediction and are unsuitable for detecting disease spots of varied sizes. On the other hand, one-stage methods directly transform the object localization problem into a regression problem, such as YOLO [21] and SSD [22]. However, YOLO lacks regional proposals and does not fully leverage local information.

The SSD algorithm is designed to achieve accurate and fast target detection by incorporating a multiscale prediction mechanism that utilizes features of varied sizes for classification and regression. Wang et al. [23] improved this by adding a coordinated attention module with a global attention mechanism to the dense block, thereby enhancing the accuracy of target localization and identification. Additionally, they replaced the Inceptionv2 module in the first three additional layers of the SSD structure to strengthen the model’s capability to retrieve feature information. Furthermore, the outputs of each additional level are fused with the sublevel outputs through convolution and pooling operations, enabling the integration of image feature information across different levels. These enhancements in the SSD-based object detection algorithm contribute to improved target localization and recognition accuracy. Another improvement was proposed by Zhichao et al. [24], who selected the MobileNetv2 network as the backbone feature extraction network and incorporated the channel attention mechanism for feature weighting. Gao et al. [25] suggested replacing the VGG16 network with the ResNet50 network as the backbone network framework and increasing the input resolution of the model to extend its detection range. However, it should be noted that the shallow layers still lack sufficient information on small targets, and the features in the small target region may appear smaller than their actual size when mapped back to the original image.

This study aimed to detect jujube black spot disease in Alar City, Xinjiang. It proposed a method called JujubeSSD for identifying black spot disease in Jujube utilizing the SSD network. Initially, a dataset with diverse samples of jujubes exhibiting diverse sizes, shapes, densities, and black spot locations was created through artificial collection and data augmentation. This dataset served for training the model. To expedite the training process, parameter information obtained from transfer learning on the COCO dataset [26] was incorporated into the backbone feature extraction network module of the SSD model. Additionally, the traditional convolution layer was substituted with DCN [27] to facilitate the acquisition of detailed target features and enhance the recognition of weak information concerning the location of jujube disease spots. Finally, PAFPN [28] and BFP [29] were integrated into the SSD network to amplify multiscale features and balance the information derived from different scales, thereby overcoming the challenge posed by the varying size and shape of the black spot region in jujube.

## Materials and methods

### Structure of JujubeSSD

**Fig 1:**
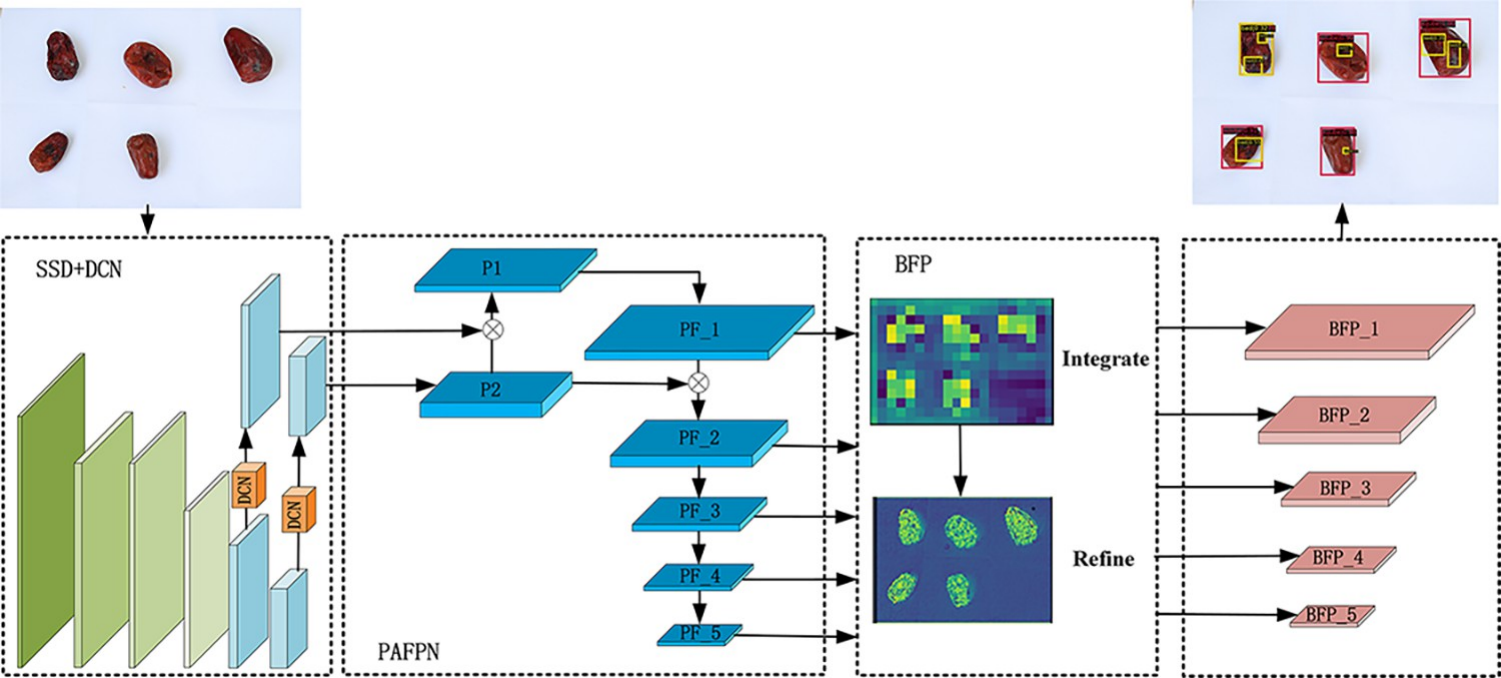
The structure of JujubeSSD.

The proposed method for detecting jujube black spots is illustrated in Fig 1, presenting a comprehensive framework. This method is composed of four essential components: data collection, replacement of the traditional convolutional layer with DCN, integration of PAFPN into the SSD network, and integration of BFP into the SSD network. Each component plays a critical role in achieving accurate and reliable disease detection.

To train the model, a diverse dataset of jujube samples with diverse sizes, shapes, densities, and black spot locations is meticulously collected and augmented. The traditional convolutional layer is substituted with DCN, enhancing the model’s ability to learn detailed target features and effectively recognize weak information regarding the location of jujube disease spots. Furthermore, the integration of PAFPN improves the representation of multiscale features and ensures balanced information exchange across different scales, effectively addressing the challenge posed by the varying size and shape of the jujube black spot region. Additionally, the integration of BFP into the SSD network results in several advantageous effects such as denoising, smoothing, feature enhancement, edge preservation, detail restoration, robustness, and stability. These effects significantly enhance the performance of the neural network, making it more suitable for several types of black spot disease recognition tasks.

Collectively, these components contribute to a robust framework capable of accurate and efficient disease detection. This method holds significant potential to augment agricultural practices and facilitate effective management of jujube cultivation in the context of black spot disease.

### Jujube black spot dataset

#### Data acquisition

**Fig 2:**
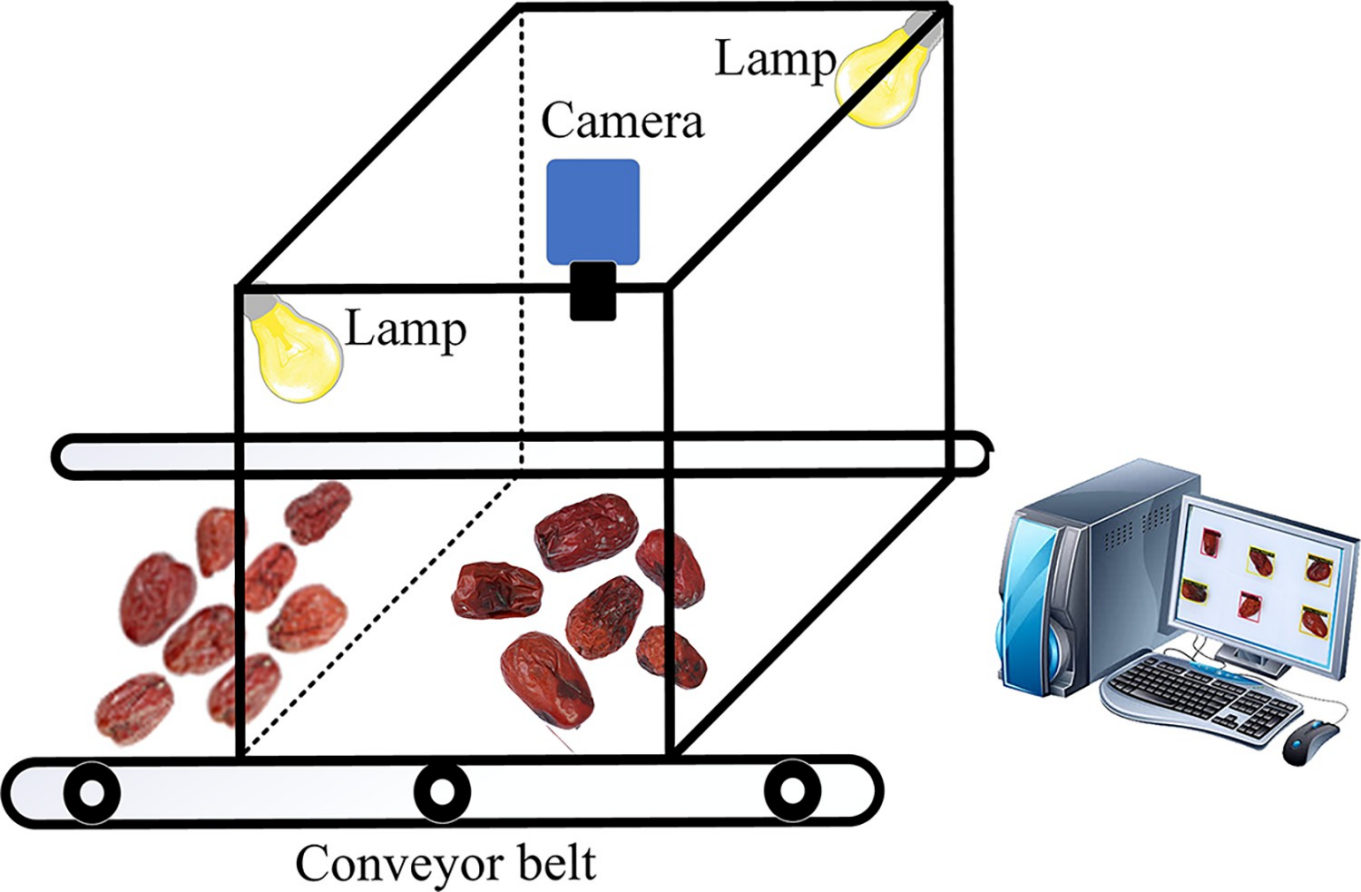
Image acquisition equipment.

This study developed a visual inspection system that emulates an actual production line to capture both static and uniformly moving jujube images. The system comprises five components: a conveyor belt, jujubes, a light source, a camera, and a computer, as depicted in Fig 2. The conveyor belt is sized at 30 cm X 150 cm and can be regulated to maintain a constant transmission speed between 4 and 15 m/min. Diverging from conventional bottom illumination methods, this research adopted two side-forward lighting techniques. Specifically, the light source is positioned above the jujubes, while the camera is located at the top. This configuration not only fulfills the prerequisites for acquiring high-quality images but also simplifies equipment installation and minimizes costs.

The image quality has a direct impact on the accuracy of subsequent detection algorithms. Accordingly, an industrial camera equipped with a 90° undistorted fixed-focus lens was utilized to capture RGB images with a resolution of 4,000 X 6,000 pixels. The sample shooting distance ranged from 30 to 60 cm. In total, 284 images (1,803 Jujubes) were acquired to form a diverse dataset, comprising various angles, and jujube conditions. This dataset included 1,062 images exhibiting signs of black spot disease and 741 images representing healthy jujubes that were not affected by the disease. Each image contained multiple intact jujubes and those affected by black spot disease, with an average of more than six jujubes per image.

The images in the black spot disease dataset were randomly divided at a ratio of 9:1 for experimentation purposes. Specifically, 1,609 Jujube images were allocated for training, while 179 Jujube images were designated for testing. The validation and test sets were derived from the same dataset. convolutional neural networks (CNNs) are widely recognized for their need for a substantial number of training images to effectively capture features. However, the available training dataset for black spot disease in jujubes comprised only 1,803 jujube images, which may be considered insufficient to train deep networks adequately. Since image data inherently possess a structure, applying specific structural transformations to the images enables the generation of data that mimic real-world scenarios not present in the original dataset.

**Fig 3:**
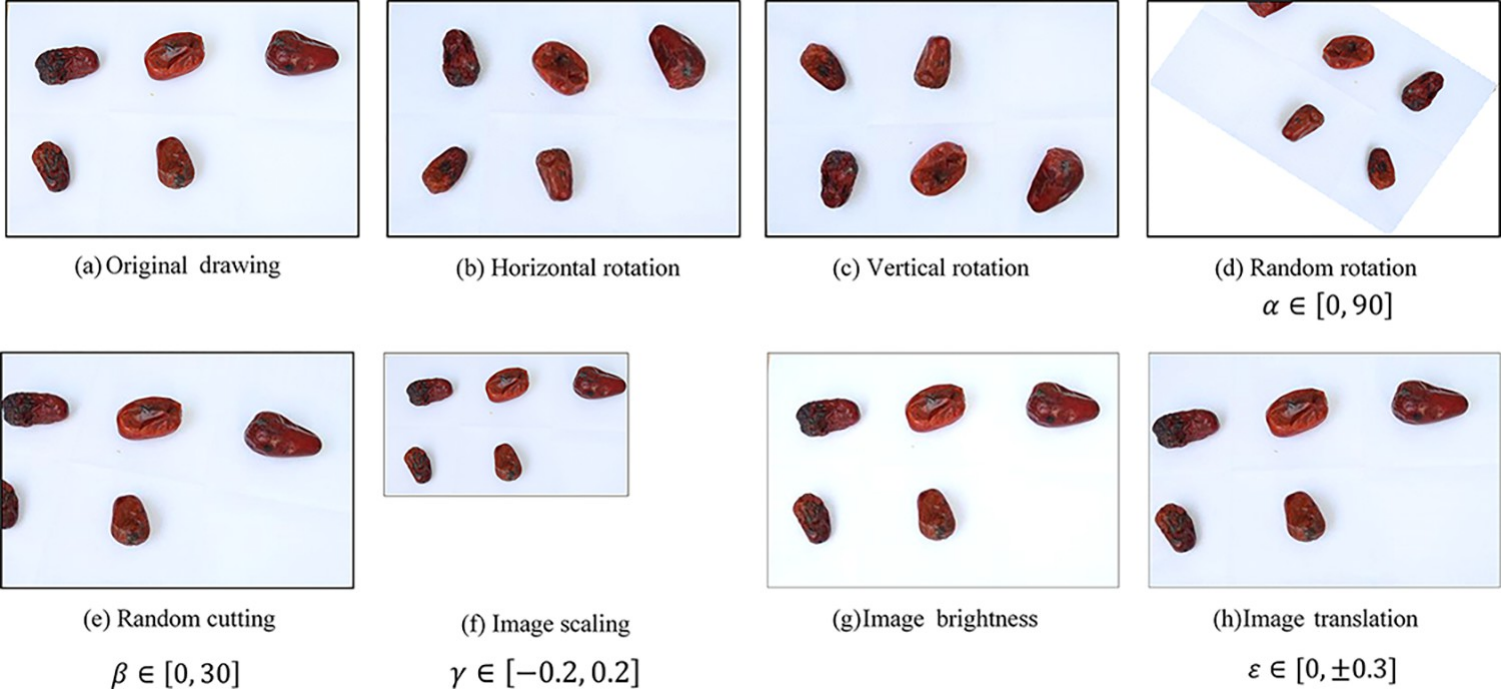
Data augmentation. (a) Original drawing; (b) Horizontal rotation; (c) Vertical rotation; (d) Random rotation with a rotation angle between 0 and 90 degrees; (e) Random cutting with a pixel value between 0 and 30 pixels; (f) Image scaling compared to the original drawing is between -0.2 and 0.2; (g) Image brightness; (h) Image translation compared to the original drawing is between 0 and ±0.3.

The process of screening jujube fruits is complex due to their extensive range of characteristics, including location, size, morphology, and texture. In addition, the lighting conditions during image data collection can vary significantly. To address these challenges, this study employed digital image processing methods and harnessed the existing image information to simulate realistic environments and generate an adequate number of training samples. Specifically, we utilized nine data augmentation techniques, resulting in the generation of 1,988 images featuring multiple black spots on jujube fruits. Fig 3 visually illustrates the process of data generation.

#### Data labeling

The manual annotation process encompasses multiple steps, including data preparation, image display, disease spot labeling, and healthy region labeling. Annotators carefully examined the images and utilized annotation tools to label the areas affected by disease spots and mark the healthy regions. Subsequently, the labeled information was linked to the corresponding images and saved. Throughout the annotation process, agricultural experts provided guidance, ensuring accurate annotation of the disease spots. For each image, five team members simultaneously performed annotations, and approval from at least three members was necessary to validate the annotations.

The process of identifying regions of interest (ROIs) in an image and precisely marking their positions using labeling software or a labeling program is commonly referred to as document labeling. In this study, we employed the visual image annotation tool LabelMe [30] for conducting manual annotation tasks. Considering the distinct features of jujube fruit and the black spots, which are small and exhibit varying shapes, we labeled them separately. The black spot areas were designated with a green border and categorized as ’bad’, while the jujube fruit areas were outlined with a red border and categorized as ’jujube’. All pertinent information, including the target species name, target location, and image location, was recorded and stored in a JSON file.

#### The features of the data blackspot dataset

The jujube black spot dataset comprises a vast collection of training samples, encompassing images of jujube fruit and black spots of varying sizes. The dataset incorporates black spot images that exhibit diverse shapes, colors, and textures, representing a wide spectrum of manifestations of black spot disease in jujube fruits. This diverse range of samples facilitates improved learning and comprehension of different types of jujube black spot disease by the model, enhancing its ability to generalize to unknown samples. Furthermore, the dataset includes comprehensive annotation information, capturing key attributes such as the position, size, and severity of black spots in each image. This detailed annotation serves as a valuable reference for further research and analysis, enabling researchers to delve into pertinent issues related to jujube black spot disease and advancing its development and application in the field.

*Experimental Materials*. The experimental site for this study was situated in Alaer, a city renowned for its extensive and densely populated jujube cultivation region in southwestern China. Field trials and data collection were conducted within a jujube orchard located at the College of Horticulture and Forestry Experimental Station’s Jujube Cultivation Base (40.548227 N and 81.305909 E) and the 10th Regiment in the Southern Xinjiang Region (40.622797 N and 81.306232 E). The selected jujube strain for this study was Junzao No. 1, with harvesting conducted on October 29th. The region experiences a warm-temperate arid climate with annual precipitation levels typically not exceeding 100 mm. In September and October, a decrease in temperature and an increase in precipitation resulted in fruit splitting among the jujube trees. Additionally, some jujube trees exhibited symptoms of black spot disease in early October. Sampling occurred at three distinct time points: the onset of black spot disease (October 15th), during the harvest period (October 30th), and one month after storage (November 30th).

*Sufficient training samples*. The dataset for jujube black spot disease comprised a total of 1,988 images, with each image containing 5~7 jujube samples, resulting in a total of 12,516 jujube samples. Among these, 284 original images encompassed 1,788 jujube samples. Following data augmentation, the dataset was expanded to 1,977 images containing 12,516 jujube samples. The training set consisted of 11,263 jujube samples, while the validation and test sets encompassed 1,253 jujube samples each. Table 1 presents the distribution of black spot samples among the training, validation, and test sets. Notably, the training set housed the highest number of jujube samples, facilitating effective learning and comprehension of the characteristics associated with jujube black spot disease. Smaller subsets of jujube samples in the validation and test sets (both 1,253) were utilized to assess the model’s generalization ability and performance.

**Table 1:** Jujube black spot dataset.

	Samples	Raw data	Data augmentation
**Training set**	Image	254	1,789
	Jujube	1,609	11,263
**Validation/ Test set**	Image	30	199
	Jujube	179	1,253
**Total**	Image	284	1,988
	Jujube	1,803	12,516

**Fig 4:**
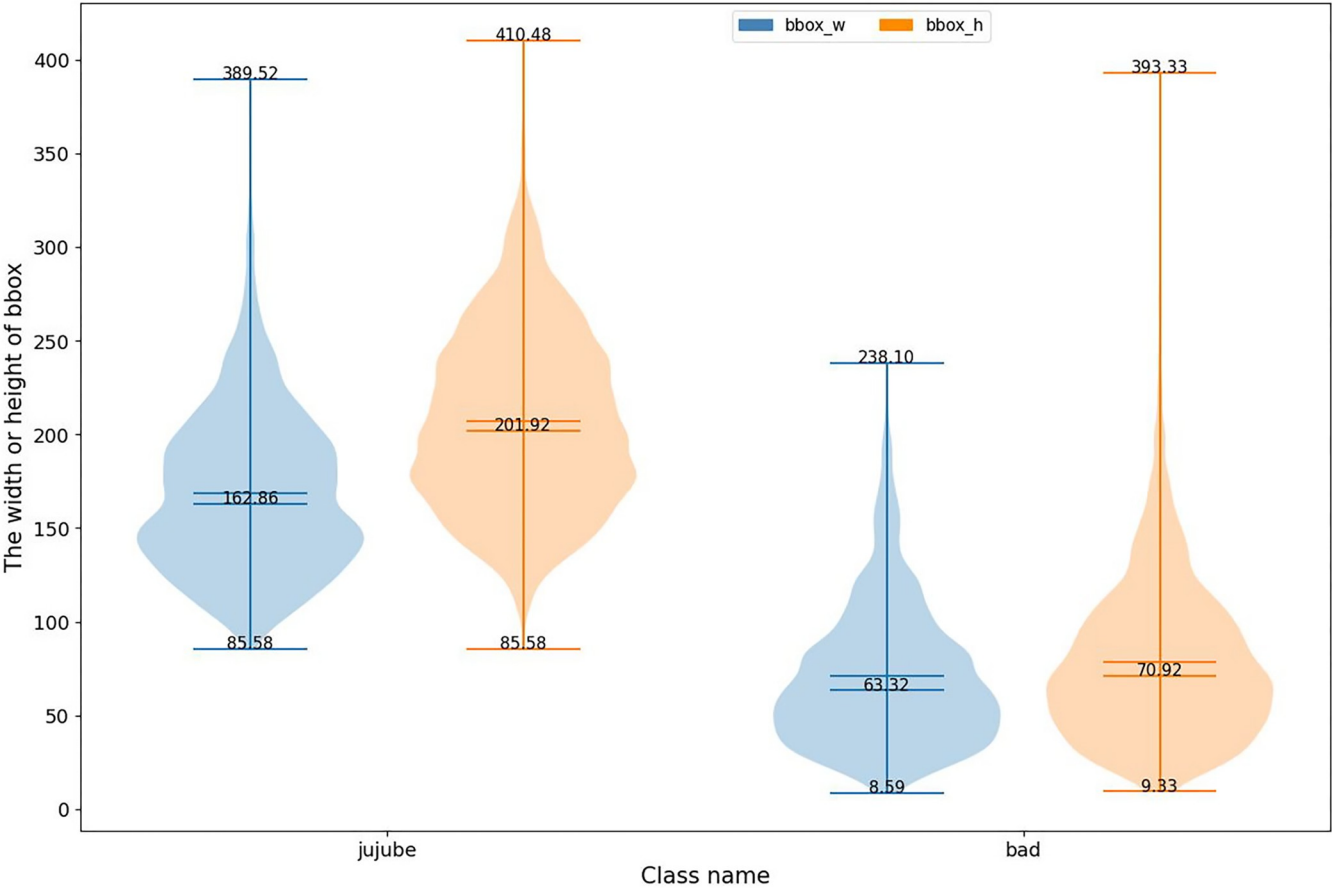
Label width and height distribution.

*Different shapes*. The black spot dataset includes Fig 4 and Table 2, which illustrates the distribution of widths and heights for both the jujube fruit and black spot areas. The widths of the jujube fruit areas ranged from 85.58 to 230, while the heights varied from 100 to 300. Similarly, the black spot areas exhibited variations in width (ranging from 8.59 to 150) and height (ranging from 9.33 to 150). These findings highlight notable disparities and divergences in the morphology and size of the annotated regions. Both the jujube fruit and black spot areas demonstrated a wide dispersion of values, indicating a lack of consistent characteristics in their morphology and size within this dataset. Consequently, developing accurate algorithms for black spot disease detection becomes challenging, necessitating adaptable feature extraction and classification methods to accommodate the diverse forms and sizes of these regions. To address this issue, it is recommended that researchers integrate flexible approaches such as morphological processing and image enhancement techniques. These techniques can enhance the morphological features of black spot regions, mitigate sample variations, and improve the accuracy and robustness of disease detection algorithms. Ultimately, this will provide valuable support for agricultural production.

**Table 2:** The total number for each class (black spot and healthy) of the dataset.

	Total	Black Spot Jujube	Healthy Jujube
**Raw Data**	1,803	1,062	741
**Data Augmentation**	12,516	7,434	5,082

**Fig 5:**
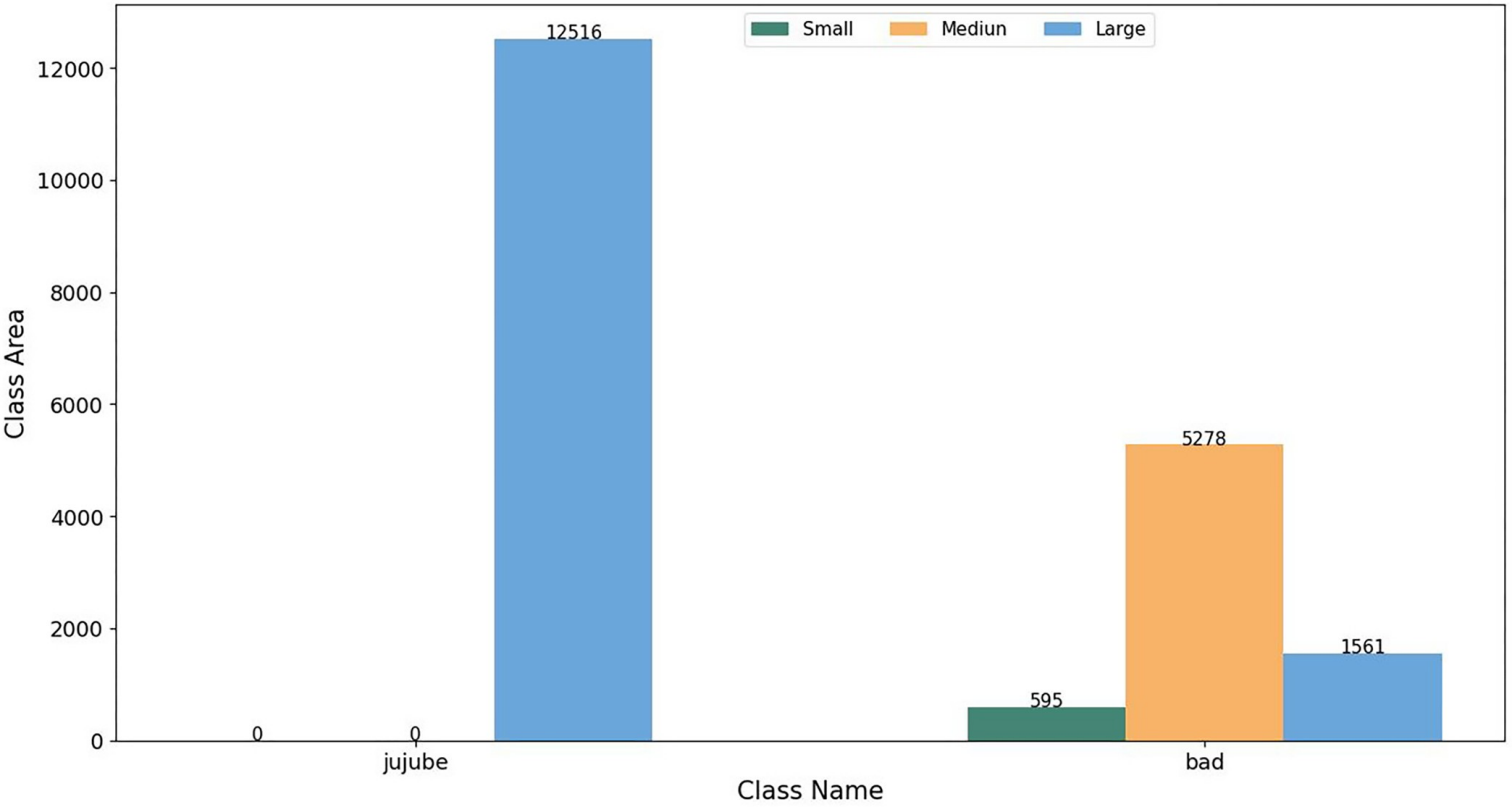
Distribution of jujube samples with black spot disease based on the size of disease spots.

*Varied sizes of fruit and disease spots*. The dataset containing jujube black spot disease comprised a total of 12,516 jujube samples, out of which 7,434 were affected by black spot disease. According to the Xinjiang jujube external quality grading standard, black spot disease was categorized into three levels based on size: small (less than 1 square millimeter), medium (greater than 1 square millimeter but less than 10 square millimeters), and large (greater than 10 square millimeters). Fig 5 illustrates the distribution of jujube samples in each category: 595 samples had small black spot disease (less than 1 square millimeter in area), 5,278 samples had medium black spot disease (ranging from 1 to 10 square millimeters in area), and 1,561 samples had large black spot disease (exceeding 10 square millimeters in area). By analyzing the Jujube black spot disease dataset, we observed that small lesions accounted for 14.2% of the black spot disease samples, medium-sized lesions accounted for 42.8%, and large-sized lesions accounted for 20.9% of the dataset.

Accurately detecting small lesions presents a significant challenge in identifying and diagnosing jujube black spot disease [31–33]. First, small lesions often manifest as tiny spots or patches on the jujube surface, exhibiting similar colors and shapes to the surrounding normal tissues, making visual observation and differentiation difficult without magnification. This poses a challenge in accurately identifying and localizing small lesions within a large-scale jujube sample set. Second, the representation of small lesions in images can be affected by image resolution and quality. Low-resolution or blurry images may obscure the details and boundaries of small lesions, leading to reduced accuracy of detection algorithms. Additionally, several factors including lighting conditions, shadows, and the texture of the jujube surface can further affect the visibility and features of small lesions in images. In this study, we improved the accurate identification and localization of small lesions by designing relevant algorithms and implementing effective data preprocessing techniques, thus providing valuable support for the sustainable development of the jujube industry.

### Transfer learning

The proliferation of deep learning techniques for image recognition has spurred researchers to explore their application in agricultural disease identification, aiming to achieve efficient and accurate results in detecting agricultural diseases [34–36]. To this end, a transfer learning strategy [37] is employed, leveraging knowledge acquired from other datasets to enhance the accuracy of disease identification and reduce detection time. In this study, we utilized a parameter-based approach to adapt a model trained on the COCO dataset for the identification of jujube black spot disease. While the COCO dataset serves as a valuable starting point, there are substantial differences between it and the jujube black spot disease dataset, necessitating adaptation for optimal results.

To address the challenges of high computational load and overfitting in the SSD model for identifying dried jujube black spots, this study proposed a solution that involved pretraining weights in the backbone feature extraction network using transfer learning. Unlike previous approaches that froze weight parameters in the VGG network, this study did not freeze any layers in the SSD backbone feature extraction network. This was because freezing more layers could reduce the model accuracy and increase overfitting. Moreover, freezing layers weakened the model’s ability to extract features from jujube black spot images, and the shared features among the layers were weakened. As a result, the model could only perform migration learning on higher-level features, and it could not gradually abstract, inscribe, or extract features from lower to higher levels. This led to a gradual decrease in the recognition rate of the model.

### DCN adaptive feature extraction network

The improved SSD method for detecting jujube black spot disease incorporates deformable convolution to adjust the weights and positions of sampling points. The shape of the black spot varied significantly, which posed a challenge when using the traditional 3 X 3 convolution sampling method. The regularity of this sampling technique often led to numerous sample points falling within the background region. As a consequence, the model’s ability to perform geometric transformations was restricted, making it difficult for the convolutional neural network to effectively accommodate objects that may exhibit different scales or undergo deformations at various locations.

**Fig 6:**
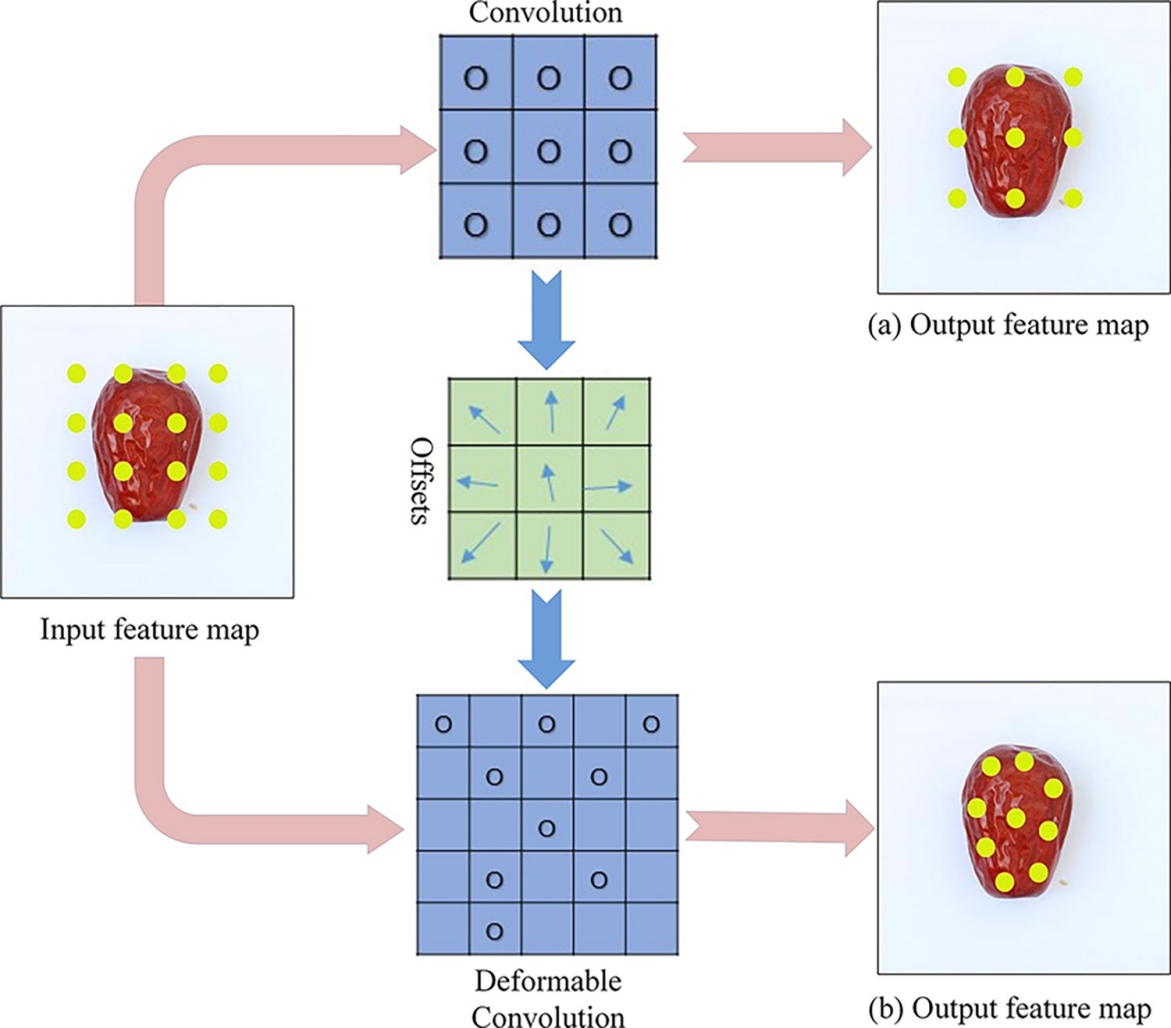
Deformable convolution introduced into SSD networks. Deformable convolutional sampling points adaptive offset. (a) Standard convolution sampling point; (b) Deformable convolutional sampling point adaptive offset.

This modification allows for a more accurate representation of the disease’s shape and is achieved by integrating deformable convolution into the SSD feature extraction network, building upon traditional convolution techniques. Previous studies have demonstrated the effectiveness of deformable convolution in various detection models [38,39]. While deformable convolution does not significantly increase the number of parameters in a convolutional neural network, using it instead of standard convolutional layers can impact inference time. To strike a balance between detection efficiency and accuracy, and to maximize the benefits of backbone migration learning, deformable convolution was integrated into the SSD algorithm. Specifically, deformable convolution was applied to the SSD neck with feature map sizes of 64 X 64 and 32 X 32 pixels. The deformable convolution introduced into SSD networks is depicted in Fig 6.

For any point on the input feature map x_i_, i = 0,⋯,n, a standard convolution y(x_i_) involves a weighted summation of the convolution kernel and input features to produce a result.


$$y(x_{i})=\sum_{x_{n}\in E}w(x_{n})∙P(x_{i}+x_{n})$$ (1)

A 3 X 3 convolution kernel is positioned at the nine positions of P(x_i_) in the input, with E={(−1,−1),(−1,0),⋯,(0,1),(1,1)} serving as the sample point. w(x_n_) represents the weight of the respective position of the convolution kernel. P(x_i_+x_n_) denotes the value of the element at position x_i_+x_n_ in the input feature map. The value of an element at position x_i_ on the output feature map is represented by y(x_i_), which is obtained by convolving the convolution kernel with the input feature map.

The traditional convolution technique relies on a fixed-size and shape-convolution kernel, which may be better suited for objects with regular shapes. However, deformable convolution is capable of sampling locations that are more in line with an object’s shape and size, enabling it to learn the distinctive features of the object itself. This feature makes it advantageous to obtain more precise information by eliminating the interference of background noise, which is not possible with the original convolution method. Deformable convolution operates on the principle of incorporating a network-learned offset that causes the convolution kernel to shift toward the sampling points of the input feature map. This shift enables the kernel to focus on a desired region or target. In contrast, deformable convolution introduces an offset for each point, which is generated from the input feature map using another convolution, as shown in Eq 2.


$$y(x_{i})=\sum_{x_{n}\in E}w(x_{n})∙P(x_{i}+x_{n}+△x_{n})$$ (2)

Where △x_n_ indicates the offset.

Bilinear interpolation is a commonly used technique for obtaining the offset pixel value as the position resulting from adding the offset is typically a decimal number and not an exact pixel point. Therefore, it is necessary to utilize this technique, which can be expressed using the following equation:

$$P(x)=\sum_{Q}max(0,1-|Q_{P}-x_{P}|)∙max(0,1-|Q_{y}-x_{y}|)∙P(Q)$$ (3)

Bilinear interpolation refers to a technique in which the value of a pixel at a particular spot x is derived by considering a weighted combination of the four neighboring pixel points {Q_11_, Q_12_, Q_21_, Q_22_} that are present on the feature map. The weight assigned to each point is determined based on its proximity to the horizontal and vertical coordinates of the interpolated point. Thus, the resultant pixel value of the interpolated point is obtained.

### Balanced pyramid network of features

This study proposed a novel approach for detecting targets using a combination of PAFPN multiscale feature fusion and BFP feature enhancement techniques. The detection of black spots on dried jujubes presents a challenge because of the varying sizes of the disease, resulting in significant differences on a large scale. The SSD model [22], a well-known target detection algorithm, utilizes multiscale feature detection to fully leverage the different feature information generated during feature extraction. However, the SSD model employs separate detection at various scales and does not integrate features with varying information, thereby limiting the utilization of low-level localization information [40,41]. Consequently, the detection effect of the model is not optimal.

PAFPN technology added a bottom-up channel to the FPN, which helped to integrate features from lower levels into higher levels and transferred localization information from lower to higher levels, thereby improving the accuracy of the detection model. However, the imbalance of feature information across different scales could affect detection performance. To address this issue, the BFP feature enhancement technique was introduced to scale down the feature sizes of different scales to a uniform size for aggregation. The aggregated and enhanced feature information was then applied to the original features, which not only enhances the original feature information but also mitigates the problem of imbalance in the use of multiscale feature information. Overall, the proposed method offered a promising solution for target detection, with improved accuracy and efficiency.

### PAFPN multiscale feature fusion

**Fig 7:**
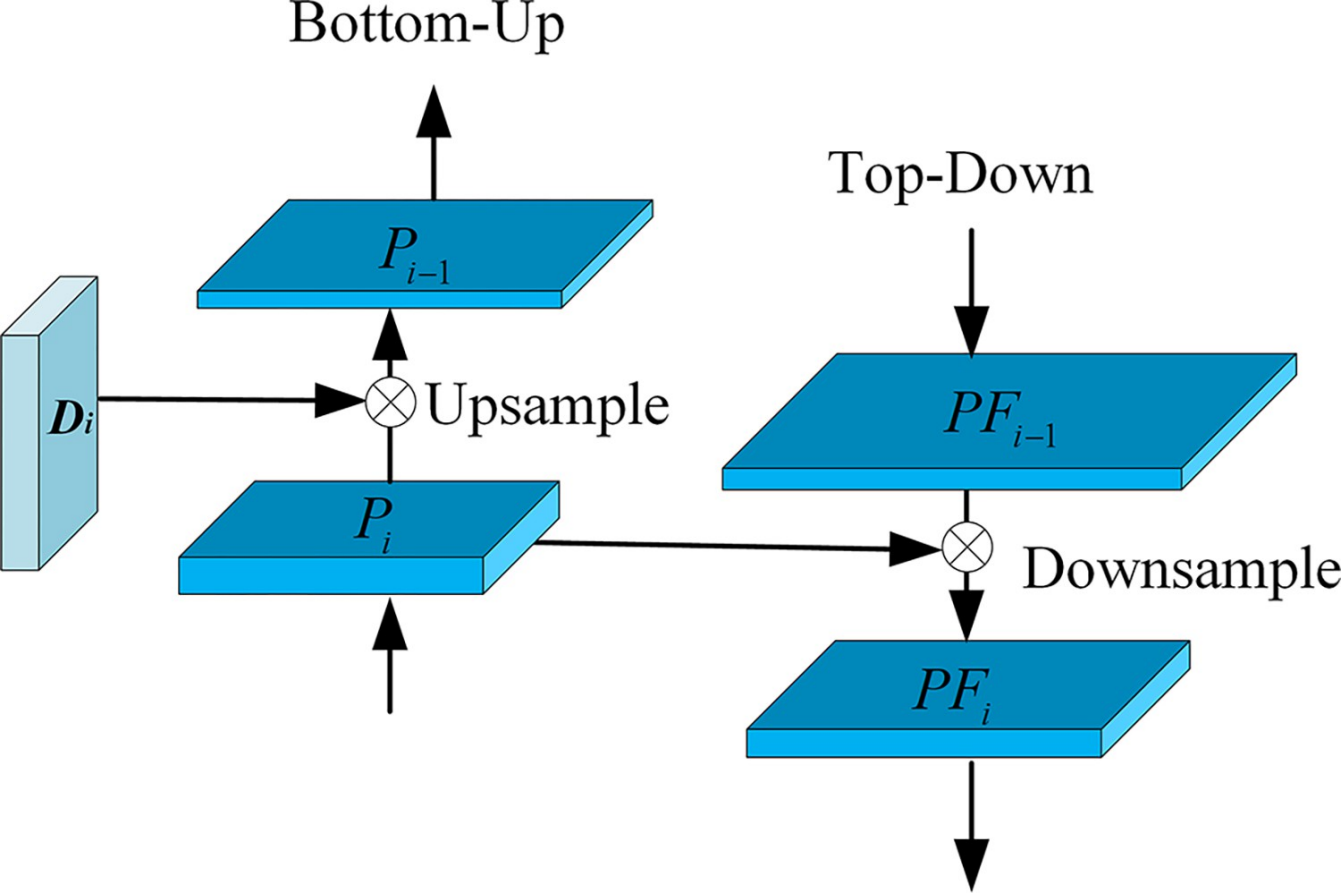
PAFPN multiscale feature fusion.

The PAFPN structure [28] is similar to the FPN structure and involves two types of feature fusion, top-down and bottom-up, as shown in Fig 7. The former transfers high semantic information, whereas the latter transfers high localization information. This structure enhances the use of the localization information at the top of the FPN. The model produces two feature maps, D_1_ and D_2_, after the SSD and DCN networks respectively, followed by PAFPN fusion. The P_2_ feature map is output by D_2_, and it is fused with the D_1_ output feature map to obtain feature P_1_. The P_2_ feature contains more semantic information, while the P_1_ feature containes more localization information. The P_1_ features are used to obtain feature PF_1_, which is then fused with the P_2_ features to obtain the feature PF_2_. This fusion process further enhances localization information based on high semantic information. In addition, PF_2_ is downsampled to generate features PF_3_, PF_4_, and PF_5_. Given an image with dimensions of 1,350 X 900 pixels, the DCN model generates a two-layer feature map that measures (512, 100 X 152) and (1,024, 50 X 76). Using PAFPN multiscale feature fusion, five distinct feature maps were produced, each with different scales and containing specific feature information. These feature maps are PF_1_ (512, 100 X 152), PF_2_ (512, 50 X 76), PF_3_ (512, 25 X 38), PF_4_ (512, 13 X 14), and PF_5_ (512, 7 X 10).

The bottom-up feature fusion path involves weighting the current layer features with all lower layers and combining them. The resulting fusion is then convolved, with a portion used as the output of the multilayer feature fusion network, and another portion down sampled and fused with the upper layers. On the other hand, the top-down feature fusion path assigns weights to the features of the current layer based on their contributions from the upper layer features. The fusion is achieved through splicing, where the weights are learnable parameters. Following convolution, a segment of the fusion result functions as the input to the bottom-up fusion path, while the remainder is up sampled and fused with the lower-layer features.


$$P_{i}^{td}=conv(cat(\omega_{1}\times D_{i}^{out},\omega_{2}\times up(P_{i-1}^{td})))$$ (4)

$${PF}_{i}^{out}=conv(cat(\omega_{1}^{\prime }\times P_{i}^{td},\omega_{2}^{\prime }\times down({PF}_{i-1}^{out})))$$ (5)

Where $D_{i}^{out}$ is the layer i feature map obtained after DCN and is used as the input feature for PAFPN processing. $P_{i}^{td}$ is the corresponding intermediate feature layer. ${PF}_{i}^{out}$ is the output of the corresponding feature layer. $\omega_{1},\omega_{2},\omega_{1}^{\prime }$, and $\omega_{2}^{\prime }$ are the weights that can be learned, up is up-sampling, down is down-sampling, and cat is the feature layer stitching operation. The weights are calculated as follows:

$$P_{i}^{td}=conv(cat(\omega_{1}\times D_{i}^{out},\omega_{2}\times up(P_{i-1}^{td})))$$ (6)

### BFP feature enhancement

**Fig 8:**
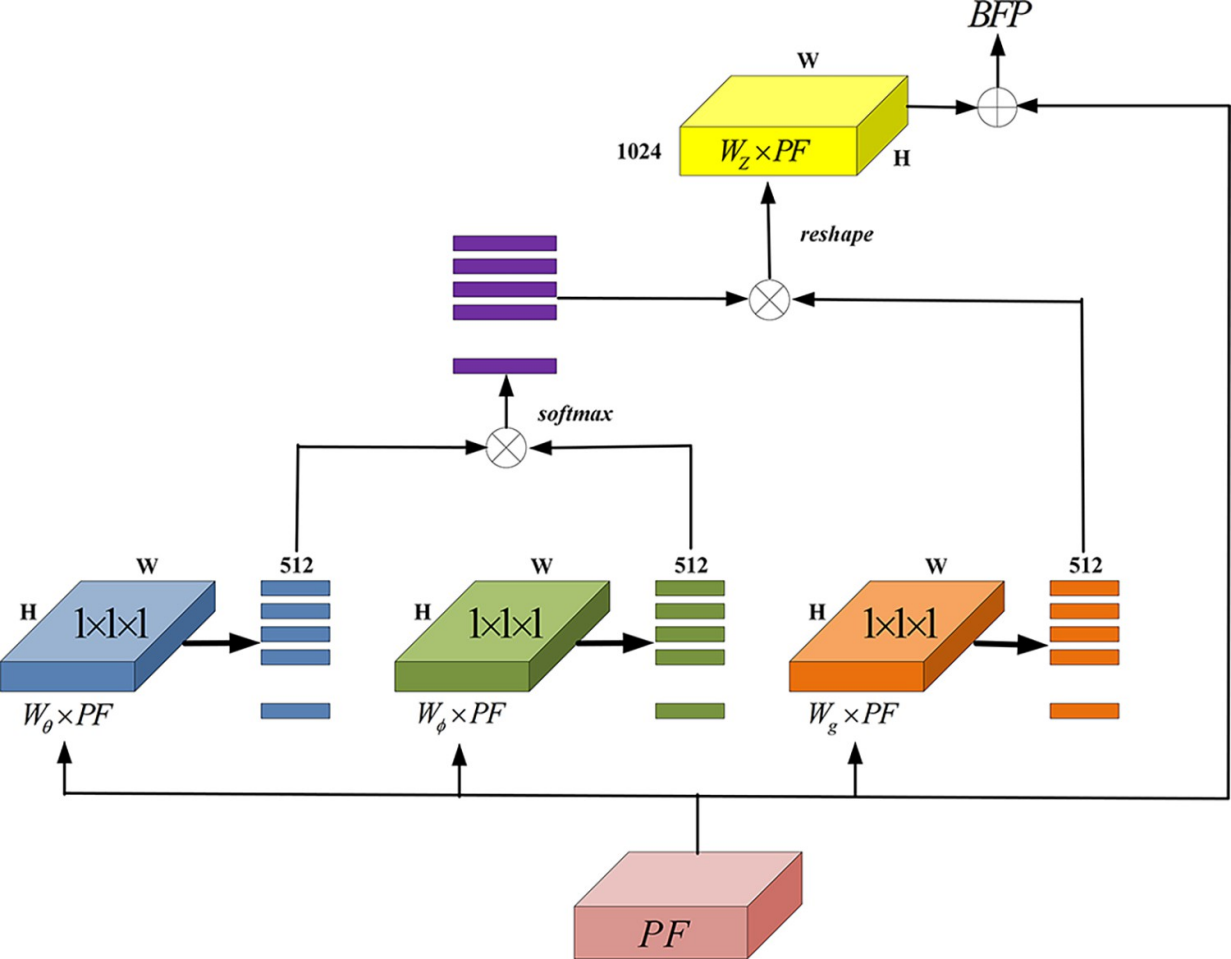
BFP feature enhancement chart.

The BFP feature enhancement method [29] is designed to improve features PF_1_ to PF_5_ and balance the multiscale feature information, as shown in Fig 8. To achieve this, the features are first scaled to the size of PF_3_ using interpolation or maximum pooling. Then, the features are integrated by averaging the scaled size information from PF_1_ to PF_5_ and the number of channels. A Refine module is then used to enhance the correlation between the pixel points in the image by modeling the aggregated features in a global context. The nonlocal structure in the refine module does not alter the input and output sizes of the features. The final features are interpolated or max-pooled to generate residual values, which are then summed with the input features PF_1_ to PF_5_ to obtain the output features BFP_1_, BFP_2_, BFP_3_, BFP_4_, and BFP_5_. The size and number of channels of the input and output features are identical. The BFP structure reinforces the input features by aggregating all features and applying aggregated reinforced features to the input features, ensuring that the input features learn equal information from the other features. The BFP achieves balanced use of multiscale feature information without altering the size of the output features. Finally, the head part of the same structure predicts and localizes targets in the image based on the BFP_1_ to BFP_2_ features. Taking the input image of 1,350X900 pixels as an example, the size of the feature maps after the multi-scale feature fusion of PAFPN are FP_1_ (512, 100X152), FP_2_ (512, 50X76), FP_3_ (512, 25X38), FP_4_ (512, 13X14) and FP_5_ (512, 7X10) respectively. After the BFP module, the output feature map size is BFP_1_ (512, 100X152), BFP_2_ (512, 50X76), BFP_3_ (512, 25X38), BFP_4_ (512, 13X14), BFP_5_ (512, 7X10).

The nonlocal module is the BFP module, which contains a self-attentive self-attention mechanism. The self-attention mechanism [42] is an attention mechanism that extends from local to global. The main idea is to focus on the information at all positions of the feature vector or feature map, mapping it to a more abstractly expressed embedding space. The main idea is to map the information to a more abstract embedding space, based on a weighted averaging-based relevance criterion, to obtain the response at any location of the image.


$${BFP}_{i}=\frac{1}{C(PF)}\sum_{\forall j}f({PF}_{i},{PF}_{j})\cdot g({PF}_{j})$$ (7)

Where PF denotes the feature map of the input BFP network, BFP represents the output feature map after the BFP calculation, i indicates the position of the input feature map, and j represents any possible position index of the feature map. C(PF) is the normalization function, and f(PF) is the attention weight factor.


$$C(PF)=\sum_{\forall j}exp({PF}_{i}^{T}\cdot {PF}_{j})$$ (8)

$$f({PF}_{i},{PF}_{j})=exp({PF}_{i}^{T}{\cdot PF}_{j})$$ (9)

g(PF_j_) is determined by the linear transformation g(PF_j_) = W_t_∙PF_j_, where W_t_ is the weight matrix obtained by convolutional learning. If PF is the corresponding feature map, t can be represented as a 1 X 1 X d_v_ convolution operation, d_v_ is the dimension of PF, which is the number of channels of the above feature map.

### Evaluation metric

**Confusion matrix [43].** Once the model is trained and target detection experiments are conducted, it is important to visually demonstrate the effectiveness of the detection frame. However, it is necessary to use quantifiable parameters to evaluate the model. For classification tasks, the predicted categories are compared with the true categories. This study utilized a confusion matrix for each category, which facilitated the expression of relationships between the detected objects. Table 3 lists the specific relationships in the confusion matrix.

**Table 3:** Confusion matrix.

		Ground Truth	
		Positive	Negative
**Predicted Value**	Positive	TP	FN
	Negative	FP	TN

The confusion matrix contains four basic elements: TP (true positive), TN (true negative), FP (false positive), and FN (false negative). TP denotes the count of targets that are positive samples and have been correctly identified as discriminatory. TN denotes the accurate identification of negative samples, which in turn indicates the absence of discrimination. FP denotes the count of targets that are erroneously labeled as positive samples. FN is the number of targets that should be positive but are classified as negative.

#### Accuracy of the network model

In terms of the accuracy of the network model, the evaluation indices are the precision ratio (P), recall ratio (R), and mean average precision (mAP). Precision is used to measure the accuracy of model checking, and recall is used to assess the comprehensiveness of model detection. The average precision (AP) is an integration of the precision over the recall, and it is indicated as the area of the region surrounded by the curve and coordinate axis in the P-R plots. The mAP means taking the average of the AP to measure the performance of the entire model. mAP∈[0, 1], the larger the value is the better the detection effect of the algorithm.


$$P_{Precision}=\frac{TP}{TP+FP}$$ (10)

$$P_{Recall}=\frac{TP}{TP+FN}$$ (11)

Where TP indicates that the jujube black spot in the tested picture is correctly recognized, FP indicates that other things are misrecognized as jujube, and FN indicates that jujube is misidentified as other things.


$$AP=\int_{0}^{1}P(x)dx$$ (12)

$$mAP=\frac{\sum_{i=I}^{N}AP_{i}}{N}$$ (13)

Where N is the number of categories, and i is the serial number.

## Results

### Detailed settings

The specific hardware configuration and experimental environment are shown in Table 4. Depending on the material conditions and the experimental values of the hyperparameters, the learning rate, the batch size, and the workers were set to 0.01, 16, and 2, respectively. The momentum factor was set to 0.937, and the decay rate of the weight was set to 0.0005. All networks were trained by stochastic gradient descent in an end-to-end way.

**Table 4:** Hardware configuration and operating environment.

Hardware	Configure	Environment	Version
**CPU**	Intel(R) Xeon(R) CPU E5-2678v3 @ 2.50GHz	OS Version	Ubuntu 18.04
**GPU**	GeForce RTX 2080Ti 10G	Framework	Pytorch 1.11
**RAM**	16GB DDR4 3200MHZ	CUDA	CUDA 11.6 Cudann 8.1.1
**Hard Disk**	BC711 512GB NVME SSD	Python	3.7

### Comparison of different object detection algorithms

#### Quantitative comparison

The model’s detection results were further evaluated and compared with other methods, namely Faster R-CNN [19], SSD [22], and YOLOv5 [44], as shown in Table 5. Notably, our proposed method achieved the highest accuracy of 97.1% in terms of mAP@0.5 on the dataset of dried jujube black spots, outperforming YOLOv5, Faster R-CNN, and SSD by 14%, 7.7%, and 5.8%, respectively. This demonstrated the superior detection accuracy of our approach. Faster R-CNN exhibited false positives due to the excessive recommendation of prediction boxes by its region proposal network, without properly suppressing duplicate target boxes through non-maximum suppression (NMS). Additionally, SSD and YOLOv5, being one-stage models, faced difficulties in detecting occluded black spots on jujubes, as their features were more complex compared to nonoccluded ones, resulting in lower feature scales and contrasts. To address these limitations, our method incorporated PAFPN multiscale feature fusion and BFP feature enhancement techniques to improve the accuracy of black spot detection.

**Table 5:** Detection results with different object detection networks.

Methods	Backbone	Precision (%)	Recall (%)	mAP@50 (%)	Detection Time (s)
**YOLOv5 [44]**	CSPNet	31.5%	45.8%	83.1%	0.03
**Faster R-CNN [19]**	ResNet	46.9%	87.0%	89.4%	0.30
**SSD [22]**	VGG	42.3%	77.9%	91.3%	0.11
**Ours**	VGG	46.0%	89.1%	97.1%	0.14

JujubeSSD is a more intricate network built on the SSD architecture, incorporating DCN, PAFPN, and BFP modules, resulting in a more comprehensive network structure. However, the detection time of JujubeSSD was higher than those of YOLOv5 and SSD. On the other hand, YOLOv5 utilized EfficientNet as its backbone network, known for its lightweight convolutional neural network structure with smaller parameters and computational size. This enabled YOLOv5 to achieve faster inference speeds while maintaining high accuracy. The longer detection time of Faster R-CNN was attributed to its two-stage object detection approach, involving dense sliding window operations and complex feature extraction. Additionally, the algorithm managed a large number of candidate boxes, leading to increased computational time.

#### Qualitative comparison

**Fig 9:**
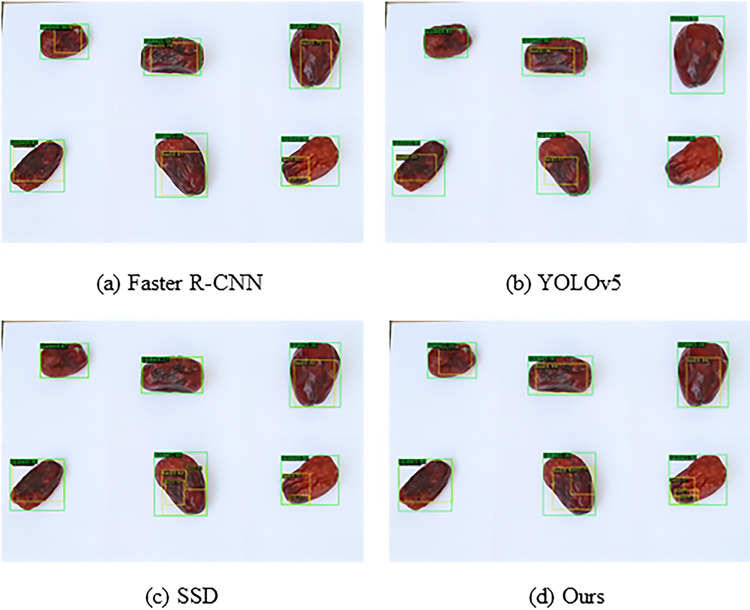
Comparison of spot detection results for different methods. (a) Faster R-CNN, (b) YOLOv5, (c) SSD, (d) Ours.

The performance of the proposed method was evaluated by comparing it with four state-of-the-art object detection networks: SSD [22], Faster R-CNN [19], YOLOv5 [44], and JujubeSSD. The intersection over union (IoU) threshold for detection in SSD was set to the default value of 0.5. All four object detection networks exhibited proficiency in accurately detecting jujubes, as indicated by the green boxes in Fig 9. However, Faster R-CNN generated multiple detection boxes in the same central region, and both YOLOv5 and SSD showed some missed detections for black spot diseases as denoted by the yellow boxes. YOLOv5 employed image size compression to increase detection speed, but this approach introduced distortions during image restoration. It failed to detect black spot diseases in three small jujubes. However, it achieved a high recognition rate for black spot diseases in medium objects. While SSD effectively detected most of the correct targets, it displayed low confidence in small targets and the black spot region in the lower-middle position. Moreover, multiple detection boxes were produced in the same central region. Due to its two-stage detection method, Faster R-CNN demonstrated slightly higher accuracy than other models, albeit at a slower detection speed.

In comparison, the JujubeSSD black spot disease object detection framework leveraged its multiscale capabilities and incorporated transfer learning with extracted convolutional features to enhance feature extraction accuracy. During testing, the proposed method demonstrated exceptional performance in detecting small target black spot diseases.

#### Confusion matrix comparison

**Fig 10:**
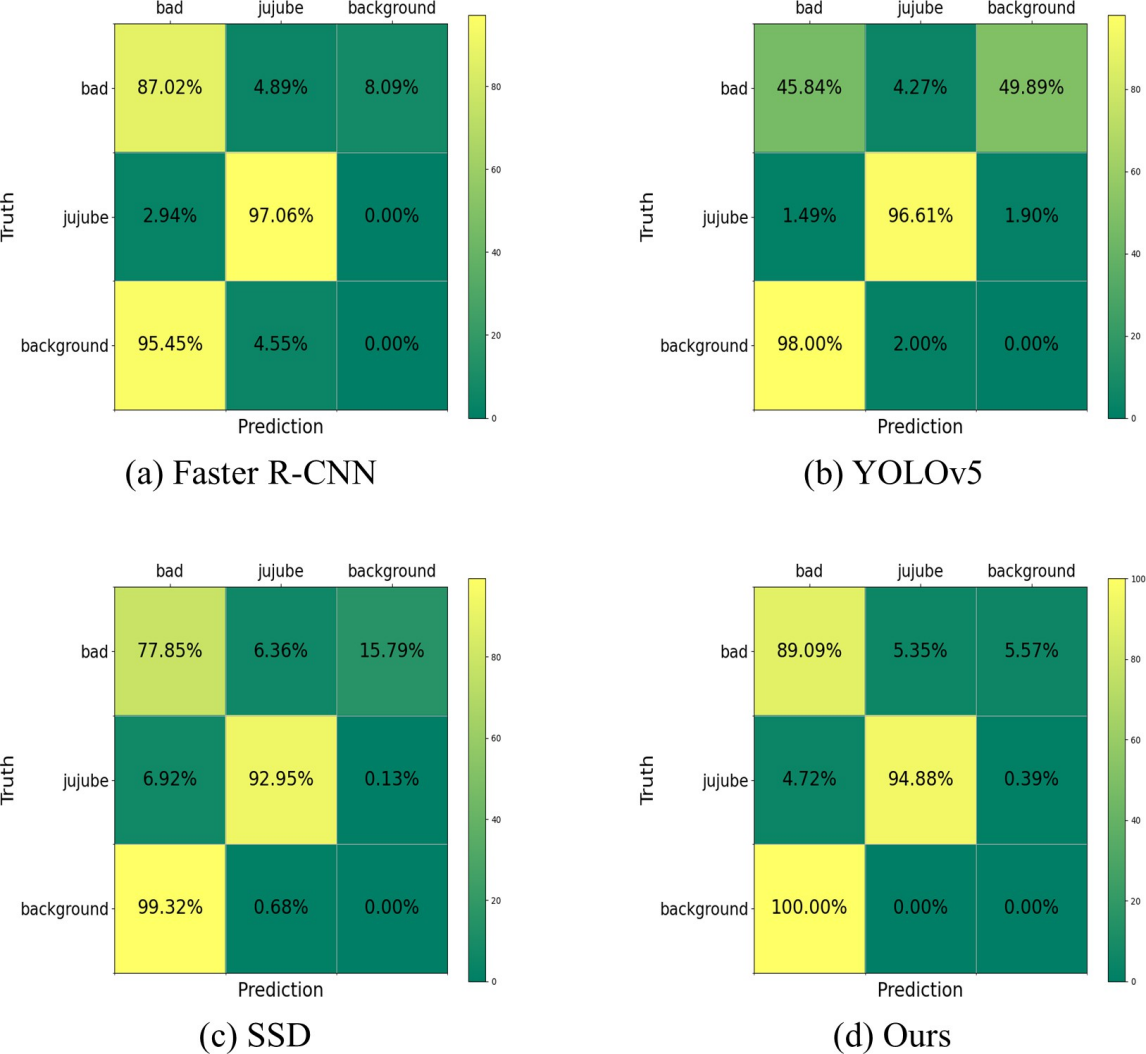
Comparison of confusion matrices for different methods. (a) Faster R-CNN, (b) YOLOv5, (c) SSD, (d) Ours.

The confusion matrix provides a detailed overview of the detection accuracy for both categories in the dried jujube black spot dataset across all comparative experiments, as shown in Fig 10. Faster R-CNN [19] demonstrated high recognition accuracy for large and medium-sized objects, albeit at a slower detection speed. It achieved the best recognition performance for large jujubes but failed to effectively utilize feature extraction for small black spot disease areas with a limited dataset. On the other hand, YOLOv5 [44] stood out for its fast speed and excelled in recognizing medium and large-sized objects, comparable to Faster R-CNN. The comparison between the SSD [22] model and the proposed model has been described earlier and is not reiterated here. In our proposed model, the black spot regions were correctly predicted in 89.09% of the cases, which was the best result among all models and the primary objective of this research. However, our model lagged Faster R-CNN and YOLOv5 in predicting the jujubes themselves. This was because, when the entire jujube constituted a large area or the entire region was a black spot area, the repeated identification of individual targets needed to prioritize recognizing them as black spot regions rather than as jujube regions. Nonetheless, our model complied with real-life production requirements and could help improve the accuracy of black-spot detection in dried jujubes.

### Ablation studies

#### The effectiveness of each module

The efficacy of three modules, namely migration learning, variable convolution addition, and feature pyramid balancing, was verified in this study. The SSD model served as the basis, and the three enhancement modules were gradually incorporated. The modified model was then retrained and evaluated, yielding the final performance comparison. Table 6 presents the ablation schemes, with the original SSD model and JujubeSSD representing the overall improved model proposed in this study. The results demonstrate that JujubeSSD is an efficient and accurate method for detecting jujube black spot disease. Compared to other methods, JujubeSSD achieved a significant improvement in the detection accuracy of black spot diseases. Additionally, the detection time of JujubeSSD was effectively controlled, making it suitable for practical production environments.

**Table 6:** Improved network ablation experiments.

Model	PRE	DCN	BFP	mAP@50 %	Detection Time s
**SSD**	X	X	X	91.3%	0.11
**SSD+PRE**	√	X	X	95.8%	0.11
**SSD+DCN**	X	√	X	93.3%	0.11
**SSD+BFP**	X	X	√	96.2%	0.12
**SSD+PRE+DCN**	√	√	X	96.1%	0.13
**Ours**	√	√	√	97.1%	0.14

“√” indicates that the improved method is applied in the model. “X” means that the improved method is not used in the model.

**Fig 11:**
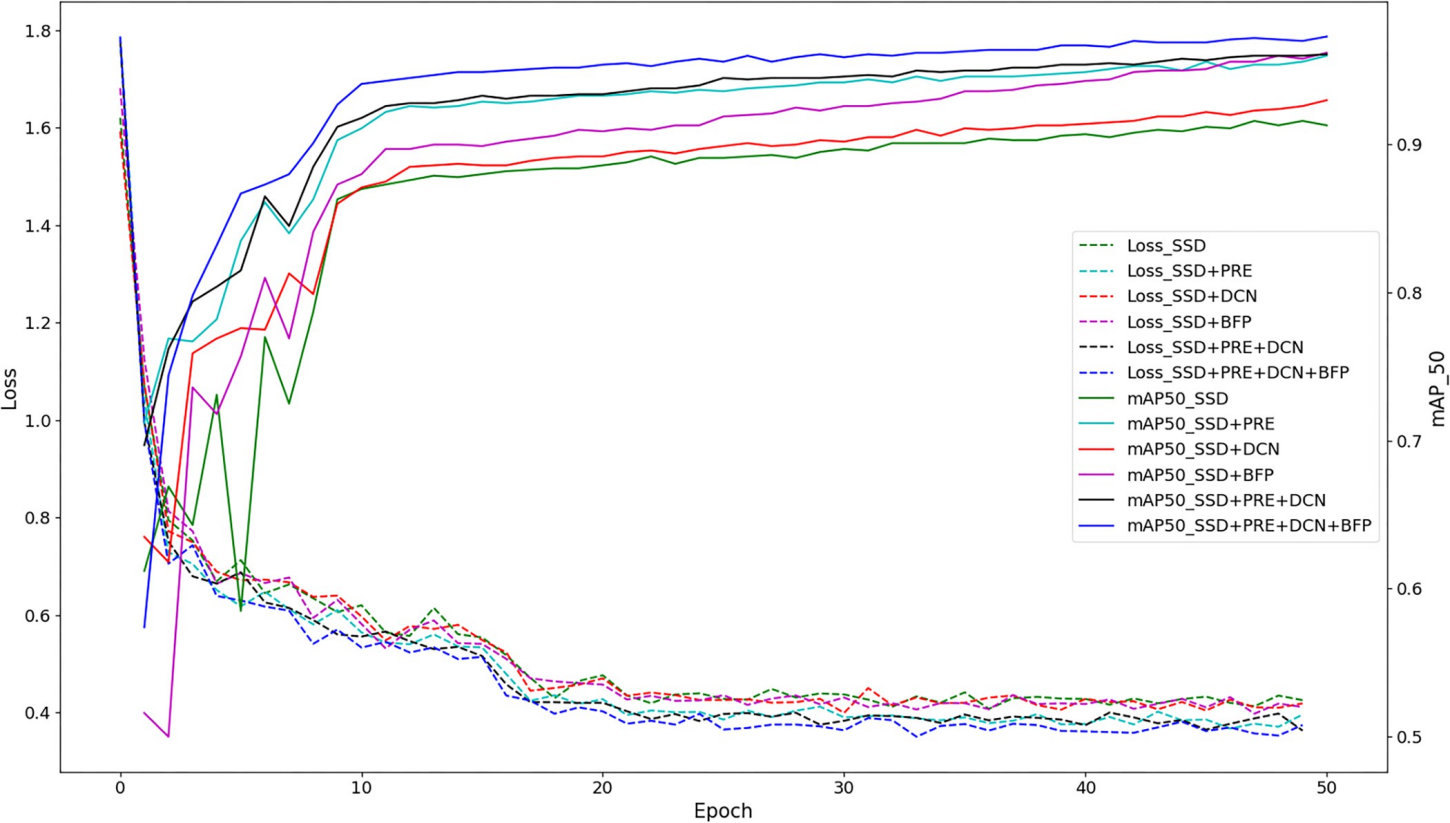
Ablation experiment training process.

The ablation experimental process is depicted in Fig 11, comprising two main parts: the loss curve graph and the mAP@50 accuracy training curve graph. A lower loss curve signified better performance, and it was evident from the graph that the SSD (baseline curve) consistently exhibited a higher loss value. Conversely, a higher mAP@50 accuracy curve indicated better performance, and SSD consistently demonstrated the lowest accuracy among all curves. All the proposed solutions exhibited higher accuracy than the baseline curve. Considering the loss curve, it could be concluded that each solution effectively improved the detection accuracy.

The experimental results demonstrated that SSD+PRE consistently exhibited significantly higher values than SSD during both the initial and final stages of training iterations. This indicated that the migration learning-based approach effectively transferred excellent empirical knowledge from the public dataset into the black spot disease model, resulting in faster convergence and improved network accuracy compared with SSD. The mAP@50 evaluation revealed that SSD+DCN outperformed the benchmark curve, exhibiting a major difference in the initial stage but gradually reducing the gap with the benchmark curve after the 10th epoch. This suggested that the DCN dynamic convolution possessed strong feature adaptiveness in the early phase, resulting in a 2% increase in the mAP@50 score for SSD+DCN on the test set. The SSD+BFP group showed slight difference in the pre-and post-improvement trends in the first one-fifth of the training process after the addition of BFP. However, as the backbone network sufficiently extracted features, the multiscale effects of feature information fusion became more pronounced, gradually distancing it from the baseline curve model. Among the schemes in which improvement modules were added separately (SSD+PRE, SSD+DCN, and SSD+BFP), SSD+BFP exhibited the most significant improvement.

Our proposed method, JujubeSSD, builds upon SSD+PRE+DCN by incorporating BFP. The inclusion of these modules resulted in significant improvements in the accuracy of the original model. However, it should be noted that the combination of these enhancement methods did not lead to a linear improvement in the performance of the new model. The evaluation metric, mAP@50, highlights that the addition of the BFP module achieved the most substantial increase in detection accuracy, effectively balancing the feature representation of the multiscale model. The second most significant improvement was observed when incorporating pre-trained weights from the public dataset, followed by the addition of DCN deformable convolution, which further aligned the extracted features with real objects. The progressive integration and comparison of these three methods yielded an accuracy increase from 91.3% to 97.1%, validating the feature extraction-based analysis and demonstrating the effectiveness of JujubeSSD in target detection for black spot disease.

#### Confusion matrix comparison

**Fig 12:**
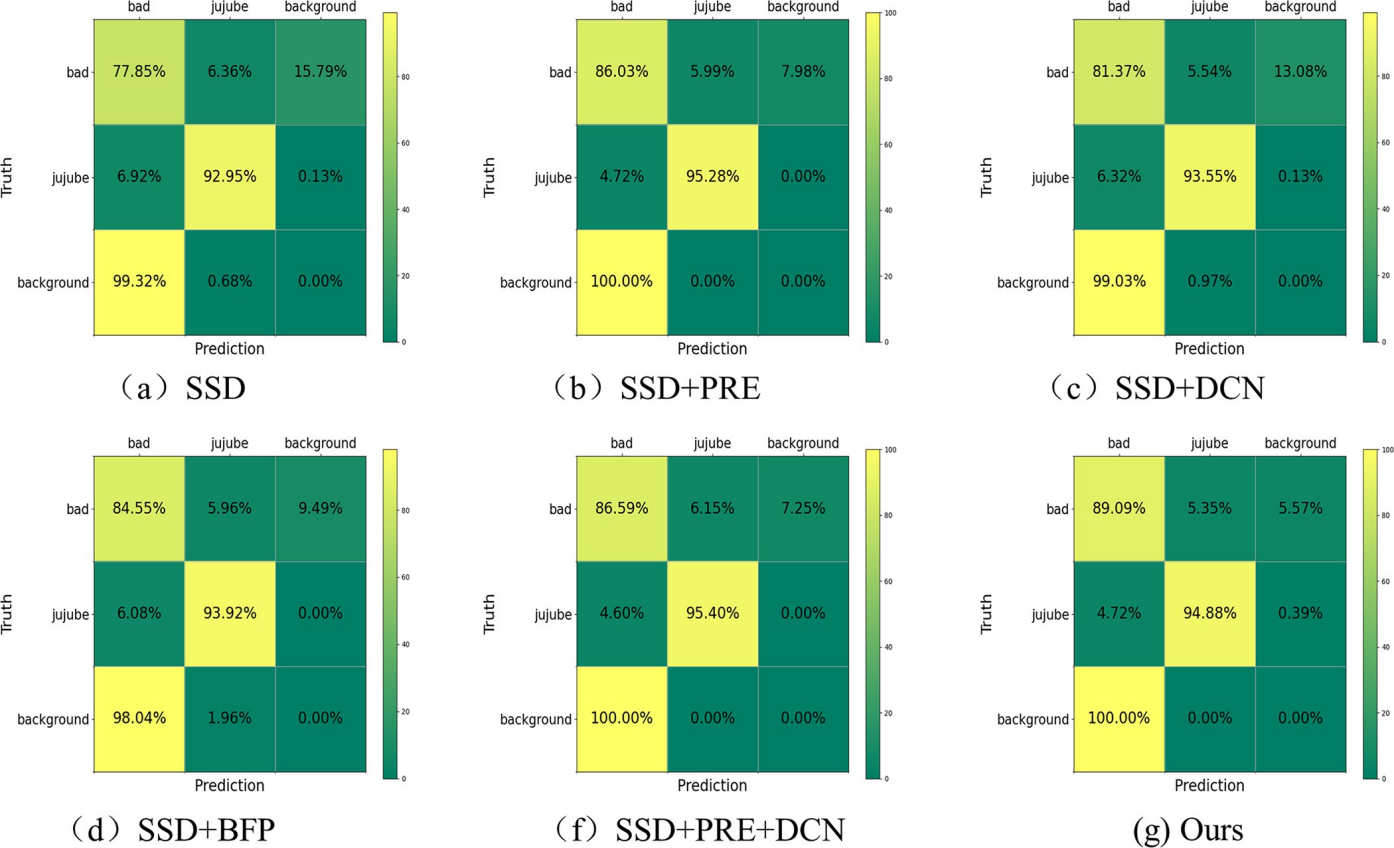
The confusion matrix comparison of the ablation experiment results.

The classification results obtained from the test set after model training are presented in Fig 12 in the form of a confusion matrix. The initial SSD model exhibited 92% accuracy in identifying features for jujubes with a large sensory field but only achieved 77% accuracy for black spots with a smaller sensory field. However, incorporating a transfer learning network resulted in a 2% increase in accuracy for jujubes with larger sensory fields and a 4% increase for those with smaller sensory fields. Notably, false predictions in the background regions decreased, as evident from the alignment between the data on black-spotted regions and the predictions of the background. Overall, the utilization of transfer learning proved effective in enhancing the detection accuracy for each category of the dataset. However, the network’s performance remains unsatisfactory for small targets, and there is room for improvement in the feature extraction capabilities of the images.

Compared to the SSD model, the SSD+DCN model exhibited a 4% improvement in precision for detecting anomalous black spot regions, as well as a 1% improvement in recognizing jujube regions despite the increase in black spot areas. This indicates that the SSD+DCN model demonstrates a notable ability to adapt to the diverse features of irregular regions. Fig 12(A) and 12(D) illustrates the results with a 10% increase in recognition accuracy for small black spot regions, highlighting the effectiveness of PAFPN multiscale feature fusion techniques and BFP feature enhancement methods in harnessing multiscale feature data and improving the detection precision of black spot targets.

The SSD model and the improved JujubeSSD target detection model was evaluated using the Jujube black spot dataset. The results showed that the improved model achieved a higher mAP@50 value of 97.1%, indicating its superiority over the baseline SSD model. The proposed method achieved a 5.8% improvement in combined accuracy detection, with mAP@50 as the primary metric. The confusion matrix per category revealed a significant improvement in the detection accuracy for small targets, with a 13% increase in accuracy for the black spot disease category. These results validate the effectiveness of the proposed method for small target detection.

### Positioning error evaluation

Our method was employed to verify the positioning accuracy by obtaining the X-coordinate and Y-coordinate of the Junzao jujubes. To depict their relative spatial relationships, six jujubes were randomly selected and positioned at grid locations on calibration cardboard with a grid size of 30 mm X 30 mm. The relative positioning errors were calculated by measuring the distances and angles between jujubes. This evaluation enabled us to assess the accuracy and precision of the method in capturing the spatial relationships among the jujubes. The predicted jujube coordinates (X_Pi_, Y_Pi_) obtained using the proposed method are listed in Table 7. The corresponding real distances in the X, and Y-directions (X_gi_, Y_gi_) of the two targets could be obtained using the calibration board. The average positioning errors (APE) can be computed using Eqs (14)-(16).


$${\Delta X}_{i}=|X_{pi}-X_{gi}|\;{\Delta Y}_{i}=|Y_{pi}-Y_{gi}|\;i=1,2,3,4,5,6$$ (14)

$$X_{APE}=\frac{\sum_{i=1}^{6}\Delta X}{6}$$ (15)

$$Y_{APE}=\frac{\sum_{i=1}^{6}\Delta Y}{6}$$ (16)

**Table 7:** Error statistics between predicted and true positions.

Samples	*X*_*pi*_	*X*_*gi*_	Δ*X*_*i*_	*Y*_*pi*_	*Y*_*gi*_	Δ*Y*_*i*_
***i*** = **1**	83	82	1	215	216	1
***i*** = **2**	503	503	0	667	667	0
***i*** = **3**	1020	1020	0	1240	1240	0
***i*** = **4**	7	2	5	191	192	1
***i*** = **5**	502	505	3	662	662	0
***i*** = **6**	1049	1050	1	1287	1286	1
***i*** = **7**	215	216	1	319	320	1
***i*** = **8**	667	667	0	321	322	1
***i*** = **9**	1240	1240	0	354	355	1
***i*** = **10**	191	192	1	776	776	0
***i*** = **11**	662	662	0	836	836	0
***i*** = **12**	1287	1286	1	755	753	2
*X*_*APE*_			1.08	*Y*_*APE*_		0.67

Where Δ*X*_*i*_ is the deviation in the X direction. X_APE_ is the average positioning error in the X-direction. Y_APE_ is calculated to be the same as X_APE_. As shown in Table 7, X_APE_ and Y_APE_ were 5.8 mm and 5.4 mm, respectively, which met the accuracy requirements for locating jujubes.

## Discussion

### Jujube black spot disease detection

With the continuous improvement and optimization of convolutional neural network models, significant progress has been made in object detection algorithms based on convolutional neural networks. Single-stage object detection algorithms, such as the YOLO series [21] and SSD series [41], are known for their fast speed but relatively low accuracy. In contrast, two-stage object detection algorithms exhibit higher accuracy but slower speed. Two-stage object detection models have shown a higher detection accuracy in the field of crop disease detection. Natarajan et al. [45] developed an automated mechanism for disease detection in cultivated lands. Deep learning techniques, specifically utilizing the deep detector Faster R-CNN and deep feature extractor ResNet50, were employed to detect and classify diseases in tomato plants. Wang et al. [46] utilized common transfer learning feature extraction networks, including ResNet50, InceptionV3, VGG16, and MobileNetV3, and pre-trained the SSD network to compare and analyze the classification accuracy and efficiency of each model. Arsenovic et al. [47] proposed a PDNet detection network and compared it with Faster R-CNN, YOLOv3, and SSD on their self-built disease image dataset, demonstrating that the proposed PDNet detection network achieved the best performance.

Single-stage object detection algorithms directly generate region proposals and predict the class of objects, thereby providing an end-to-end process. Research on single-stage object detection has mainly focused on the use of improved SSD or YOLO algorithms for crop disease detection. Compared with the YOLO algorithm, the main difference of the SSD algorithm lies in its direct detection through convolutional kernels. The SSD algorithm utilizes multiple convolutional layers for output predictions, regression, and classification at each layer. For instance, Wang et al. [48] proposed three plant leaf detection methods, squeeze-and-excitation SSD (Se_SSD), deep block SSD (DB_SSD), and DBA_SSD. Se_SSD fused the SSD feature extraction network with the attention mechanism channel, DB_SSD improved the VGG feature extraction network, and DBA_SSD combined the improved VGG network with the channel attention mechanism.

Based on the above, it can be observed that crop disease detection algorithms based on single-stage object detection exhibit high detection accuracy, real-time capability, and end-to-end optimization, making them suitable for real-time crop disease detection. However, single-stage object detection algorithms may have limitations in detecting diseases in complex backgrounds, such as cases involving high density, varying sizes and shapes of the diseases, and weak location information. In this study, we embedded DCN, PAFPN, BFP, and applied transfer learning into the classical SSD (Single Shot Multi-Box Detector) algorithm to address the challenges of high black spot density, diverse sizes and shapes of the lesions in jujubes, thus improving the accuracy of jujube black spot disease detection.

### Comparative analysis of different anchor frame models

**Fig 13:**
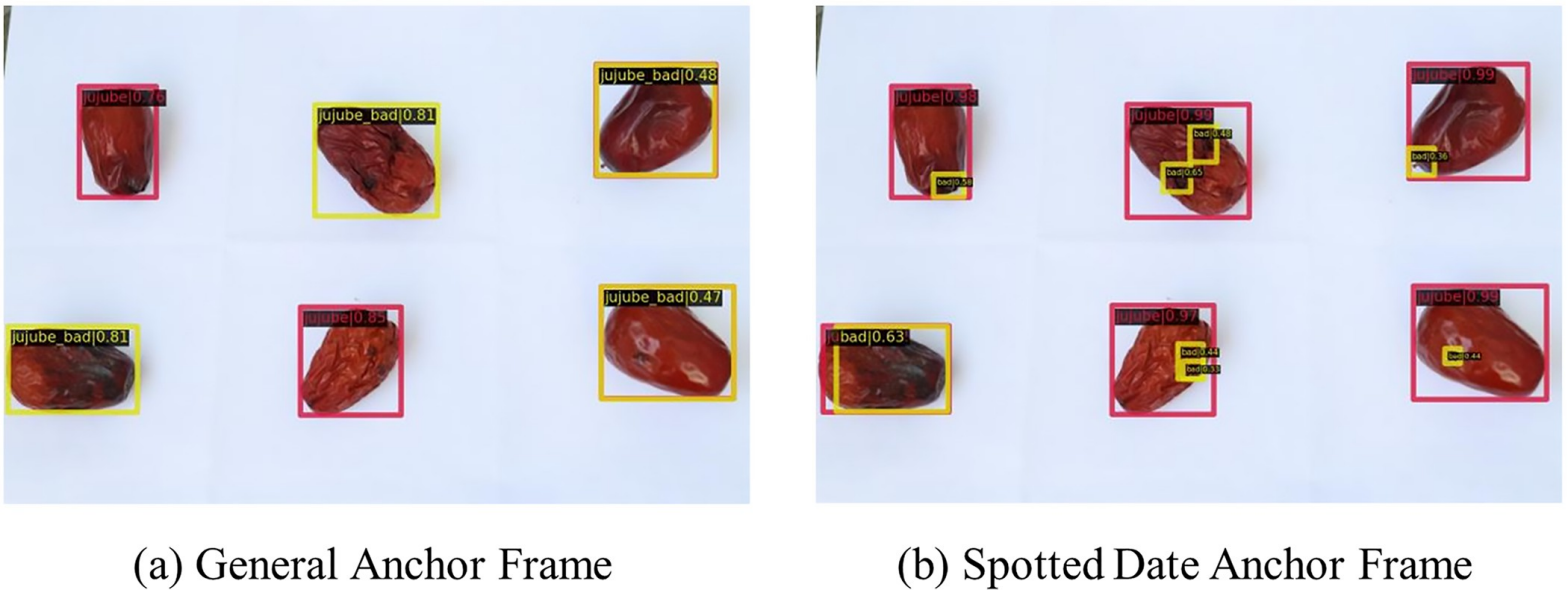
The confidence level of jujubes under different anchor frames.

The visualization plots for the general anchor frame and the spotted jujube anchor frame were compared, revealing that four out of the six stepped jujubes were correctly detected with a confidence level of approximately 80% for each stepped jujube, as shown in Fig 13. The general anchor frame exhibited a high confidence level of approximately 99% for large jujubes, demonstrating the feasibility of the general method for detecting black spots on jujubes. However, the confidence level for detecting jujubes without black spots was low, leading to potential confusion in distinguishing between jujubes with and without black spots. Notably, the method proposed in this study successfully detected black spots in the first jujube located in the upper left corner and the jujube in the lower center of the frame, both containing small black spot areas that were not detected by the usual method but were identified by our method, although with a lower confidence level.

The low confidence level of the biased small targets in the SSD model can be attributed to several factors. First, the feature extraction capability of the backbone network in the SSD model is inadequate. Specifically, the VGG16-based SSD model is not sufficiently powerful for learning-light black features. Second, the black spot region of the steed jujube is nonfixed, which limits the ability of the model to extract effective features owing to the traditional convolution approach. Finally, the feature map responsible for detecting biased small targets does not fully utilize multiscale feature fusion and fails to provide accurate feature information on the location of small targets.

### Deformable convolution optimal position analysis

This study proposed a practical approach to enhance the SSD model for detecting black spot disease in jujube. The method involved integrating a deformable convolution module into the model, which could effectively learn the spatial deformations of objects. Two embedding techniques for deformable convolution were tested to assess their impact on the detection of black spot disease in jujube. The study consisted of two stages, where the original VGG16 outputs of 64 X 64 and 32 X 32 feature maps remained unchanged after applying deformable convolution. The results of the training are presented in Table 8.

**Table 8:** The accuracy of DCN varies based on the different nested positions.

	bbox_mAP %	bbox_mAP@50 %	bbox_mAP@75 %
**SSD**	73.12%	91.32%	74.82%
**SSD+DCN1**	85.15%	92.11%	77.95%
**SSD+DCN2**	75.91%	93.35%	78.53%

**Fig 14:**
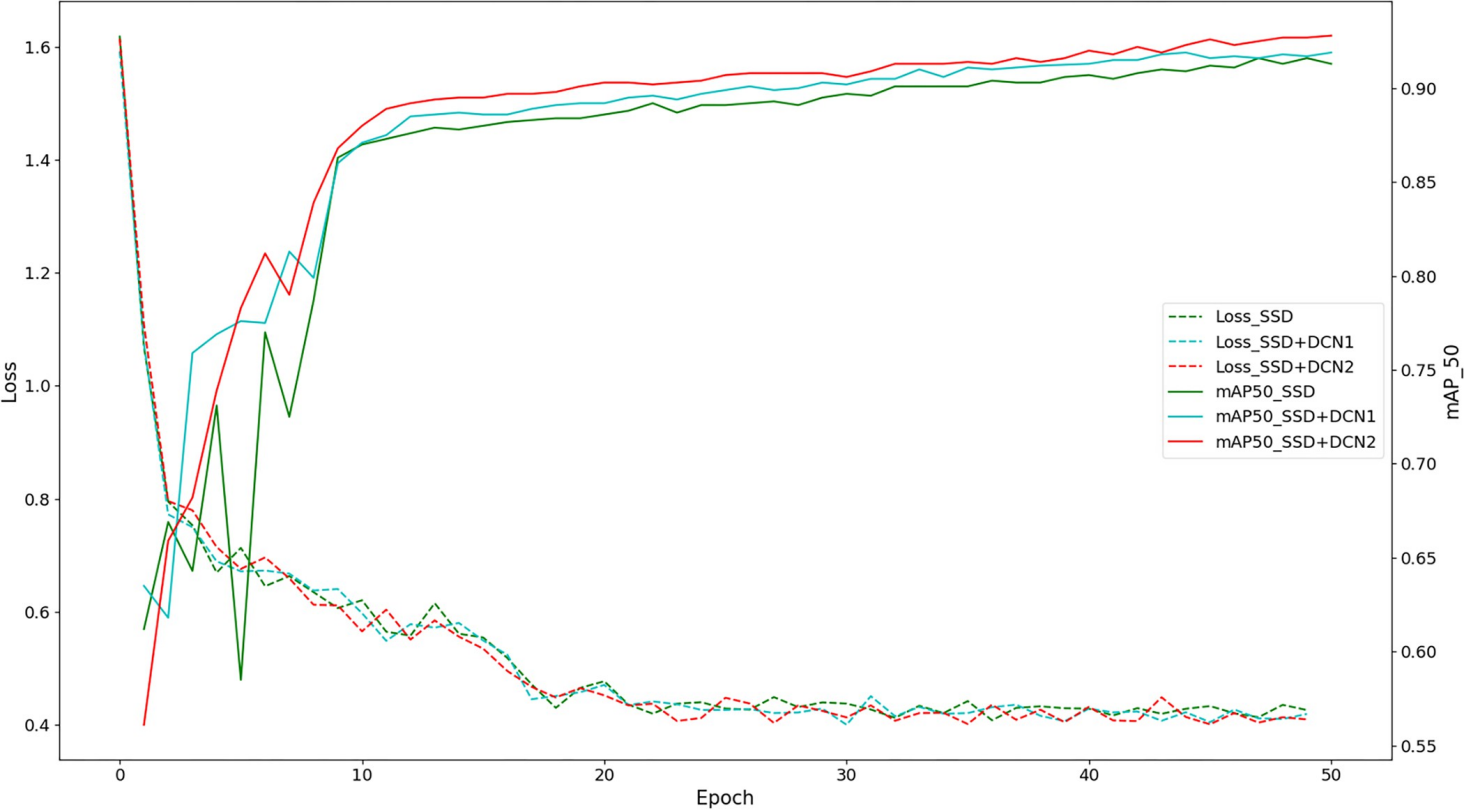
Loss and accuracy plots for deformable convolution positions.

The trends of the loss value and recognition accuracy of the model on the test set are depicted in Fig 14. Two different positions, DCN1 and DCN2, were individually chosen for embedding, and the experimental results are presented. It is evident from the figure that the SSD+DCN2 method outperforms the SSD+DCN1 method in terms of loss and test accuracy, whether measured by mAP or mAP@50 when DCN is inserted into two similar SSD positions. Notably, the results of the SSD+DCN1 method are the least satisfactory when DCN is inserted at the first position, and marginally improved when inserted at the second position. This can be attributed to deformable convolution enhancing network accuracy when the feature map size is slightly larger. However, the SSD+DCN2 method achieves slightly better results due to the relatively small number of convolutions and limited feature extraction ability of the VGG16 feature map, which has a slightly larger size and fewer feature map channels.

The results in Table 9 indicate a consistent 81% accuracy in identifying black spot disease, regardless of the embedding method used. However, the identification rate for black spot disease in stepped jujubes was higher, ranging from 93% to 94%, with a misclassification loss of 7% to 6%. It was evident that SSD+DCN2 achieved higher accuracy in terms of mAP@50, making the second embedding method the most suitable approach for this experiment. The results demonstrated that the DCN2 model outperforms the original SSD model, showing a 2% increase in mAP@50, a 4% improvement in recognizing irregular black spot disease regions, and a 2% enhancement in recognizing stepped jujube regions in the confusion matrix. These findings highlighted the adaptability of the DCN model to irregular regions.

**Table 9:** The mAP@50 values of black spot disease and jujube at different DCN embedding positions.

Method	bad	jujube	misclassification
**SSD**	77%	92%	8%
**SSD+DCN1**	81%	93%	7%
**SSD+DCN2**	81%	94%	6%

## Conclusion

In conclusion, this study presented a novel method JujubeSSD for identifying black spot disease in jujubes. The method involved embedding DCNs into the SSD network and integrating PAFPN and BFP techniques. The DCN enhanced the learning of detailed target features and improved the recognition of subtle information. PAFPN and BFP were integrated into the SSD network to enhance multiscale features and balance multiscale feature information, addressing the challenge of varying sizes and shapes of the black spot regions on jujubes. The experimental results and validation demonstrated that the proposed method provides high accuracy for black spots on jujubes. When compared to existing algorithms such as YOLOv5, Faster R-CNN, and SSD, the improvements in mAP@0.5 were 16.84%, 8.61%, and 6.35% respectively. This research contributes to the field of automated grading equipment and provides valuable insights for future work on precision agriculture technology.

In our future research, we will focus on improving several aspects. First, we aim to explore the simultaneous detection of multiple black spots in jujubes, aiming to enhance the model’s capability to detect complex patterns. Second, we plan to investigate more advanced techniques for feature extraction and representation learning, with the goal of further enhancing the model’s detection performance in specific scenarios and reducing the detection time. Additionally, we will explore the integration of state-of-the-art target detection algorithms and network architectures to further enhance the accuracy and efficiency of black spot disease detection in jujube fruits. To meet the real-time demands of the agricultural domain, we prioritize optimizing the computational efficiency and inference speed of the model. This ensures that the model can efficiently perform rapid detection in real production scenarios, making it highly practical. Moreover, we will explore additional methods for the interpretability and visualization of the model. These insights into the model’s prediction results will provide agricultural experts and decision-makers with a better understanding of its performance and comprehensive decision support.
